# Supplementary material for: Deterministic all-optical magnetization writing facilitated by non-local transfer of spin angular momentum
Source: Nat Commun. 2020 Jul 31;11:3835. doi: 10.1038/s41467-020-17676-6 (PMC7395735; doi:10.1038/s41467-020-17676-6)
Supplement: Supplementary file 1 — Supplementary Information [file 41467_2020_17676_MOESM1_ESM.pdf]

Supplementary information:
Deterministic single pulse all-optical magnetization writing facilitated by non-local transfer of spin angular momentum

van Hees et al.

SUPPLEMENTARY NOTE 1: AOS THRESHOLD FLUENCES FOR ALL MAGNETIZATION STATES

In Fig. 1(c) of the main paper we presented a measurement where we determined the threshold fluence for switching from the AP to the P state ($F_{0,\mathrm{D}}$) and vice versa ($F_{0,\mathrm{T}}$). For the sake of simplicity, we only showed data from measurements where the reference layer magnetization was in the positive direction out of the sample plane (plus-states). We assumed that, as the corresponding plus- and minus-states are time reversed versions of each other, only the relative orientation of the reference layer and free layer (P or AP) determines whether the spin current from the reference layer assists or hinders switching. Here we verify this assumption by determining the threshold fluence for switching when starting from all four possible magnetization states (P^+, AP^+, AP^-, and P^-).

In Supplementary Figure 1 we present the switched domain size in a $(Co/Ni)_{x4}/Co/Cu/Co/Gd$ sample as a function of incident laser pulse energy, when starting from all four states. The domain sizes indeed do not depend on the sample starting in a plus- or a minus-state, confirming as expected that only the relative orientation of the reference and free layer is relevant. Note that these measurements were performed on a different sample than those in

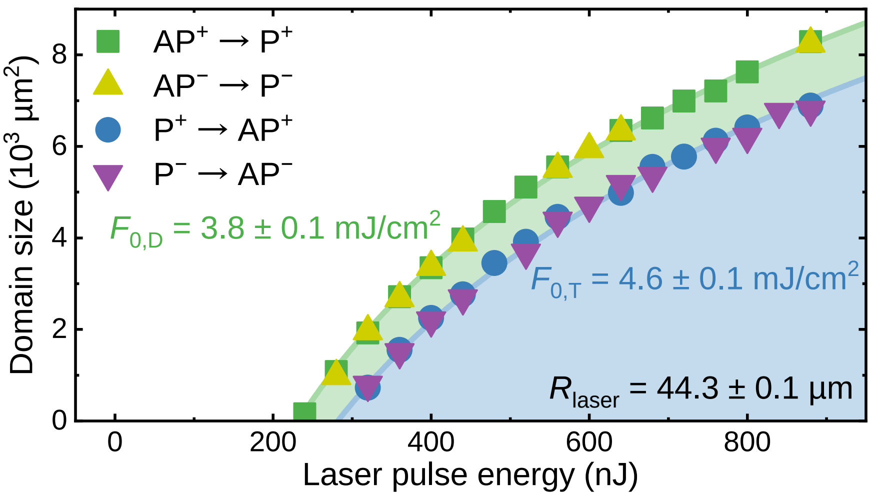

Supplementary Figure 1. AOS behaviour for all magnetization states. Switched domain size extracted from Kerr microscopy images after irradiation of a $(Co/Ni)_{x4}/Co/Cu/Co/Gd$ sample with a ∼100 fs laser pulse as a function of laser pulse energy. Fits are made to extract the threshold fluence, where each pair (P^+ and P^-, AP^+ and AP^-) is fitted simultaneously.

Fig. 1(c), yielding different values for the threshold fluence but showing the same qualitative behaviour.

SUPPLEMENTARY NOTE 2: CALCULATED OPTICAL ABSORPTION IN SAMPLES

For the analysis presented in Fig. 3(b) of the main paper, we calculated the theoretical optical absorption in the Co/Ni reference layer. As mentioned, this was done using a transfer matrix method. In this section we briefly expand on the process that was used. Using known values of the refractive index at 700 nm for all materials in the stack (from both our own measurements and literature[1]), we calculate an absorption profile of the entire stack, as shown in Supplementary Figure 2. Note that due to the likely high degree of intermixing, we treat the full Co/Ni multilayer as having the refractive index of the dominant material by volume, being Ni. The absorption in this multilayer is subsequently calculated by integrating over the thickness of this layer.

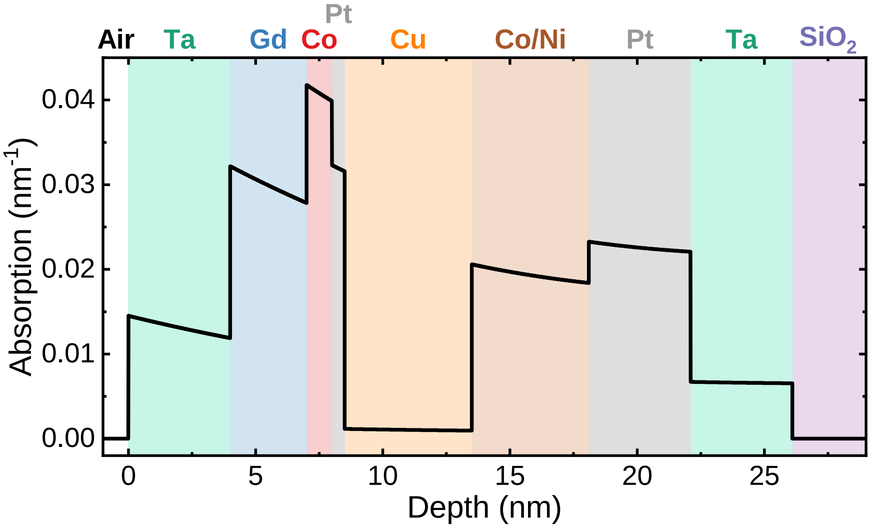

Supplementary Figure 2. Example of a calculated optical absorption profile Optical absorption per unit depth of a SiO_2(100)/Ta(4)/Pt(4)/(Co(0.2)/Ni(0.9))$_{x4}$/Co(0.2)/Cu(5)/Pt(0.5)/Co(1)/Gd(3)/Ta(4) sample as used in this work (numbers between parentheses indicate thicknesses in nm). Note that the full SiO_2 layer and Si:B substrate are included in the calculation itself.

In the same discussion we mentioned that the threshold fluences $F_{0,\mathrm{D}}$ and $F_{0,\mathrm{T}}$ were found to increase with increasing Ni thickness in the reference layer. To explain this, we posited that this could be partially explained by a difference in optical absorption in the Co/Gd layer. As the SiO_2 layer on our substrates acts as a reflective coating, the absorption is strongly affected by this reflection. Therefore, an increase in absorption in lower layers with increasing thickness could lead to a sizeable reduction of the absorption in the upper layers. To verify this, we calculated the absorption in the Co/Gd bilayer as a function of Ni thickness. We find that upon increasing the Ni thickness in each repeat from 0.5 to 1.0 nm, the optical absorption in the Co/Gd bilayer decreases by approximately 12%. At the same time, the threshold fluences increase by $\sim$19% in this same interval. The increase in threshold fluence can therefore to a large extent be attributed to the reduction in optical absorption in the Co/Gd bilayer. As mentioned in the main text, a higher roughness of the top layers could explain the additional increase, which could be verified by investigating the inverted stack (where the reference and free layer switch position).

SUPPLEMENTARY NOTE 3: EFFECT OF PT INSERT BETWEEN REFERENCE AND FREE LAYER

In the main text we showed that the difference between threshold fluences can quantitatively be explained by the behaviour of an optically generated spin current by tuning the reference layer. As a less quantitative, but more straightforward check that the effect is driven by a spin current we show a different approach here.

Following Iihama et al.[2], in Supplementary Figure 3 we present results of an experiment where we determine the difference between the two threshold fluences as a function of the thickness of a Pt insert layer between the reference layer and free layer. Note that we plot the total Pt thickness in the spacer, as a 0.5 nm Pt buffer layer on top of Cu is always included to induce PMA in the Co/Gd bilayer. It is clear here that the threshold fluence gap goes to zero within $\sim$2.5 nm of total Pt thickness. From a fit of the data with an exponentially decaying function (solid line) we extract a characteristic decay length, the Pt spin diffusion length, of (0.9 ± 0.3) nm, which is consistent with literature reports[3].

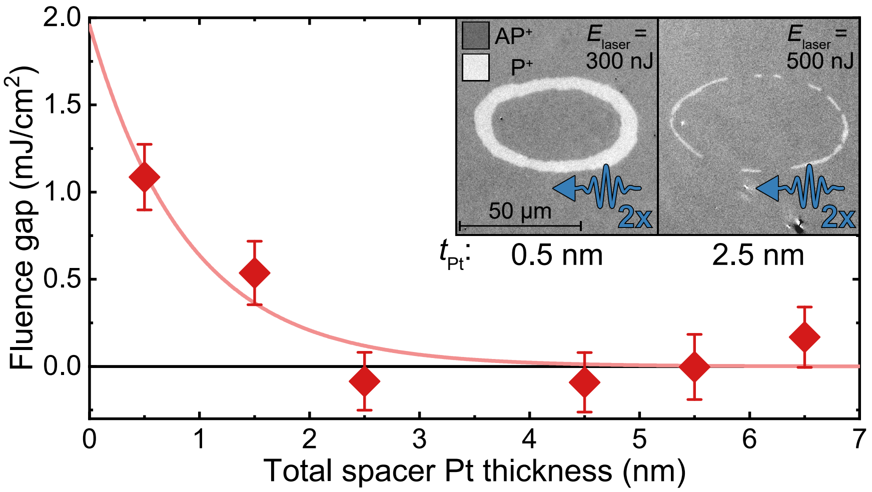

Supplementary Figure 3. Blocking the spin current with a Pt buffer. Difference between threshold fluences for deterministic writing and toggle switching as a function of the total Pt thickness between the reference and free layer in a SiO_2(100)/Ta(4)/Pt(4)/ $(Co(0.2)/Ni(0.9))_{x4}$/Co(0.2)/Pt(X-0.5)/Cu(5)/Pt(0.5)/Co(1)/Gd(3)/Ta(4) sample. Line indicates a fit with an exponentially decaying function. Inset shows the same experiment as presented in the inset of Fig. 3(a) of the main paper for two different Pt thicknesses in the spacer layer. Error bars represent the standard deviation obtained through fitting.

This is also demonstrated in the inset of Supplementary Figure 3, where we show the same type of experiment as presented in the inset of Fig. 3(a) of the main paper. There, we exposed a sample prepared in the AP state to two subsequent laser pulses with the same energy. We have already seen that this results in a ring-shaped region where the second pulse does not switch the free layer again, due to the difference in threshold fluences. Here we additionally perform this experiment on a sample with an added Pt layer of 2 nm between the reference layer and the Cu spacer layer. It can be seen that no clear ring appears, as is to be expected when there is no difference in threshold fluences. The slight broken ring which remains is the result of pulse-to-pulse variations of the laser, as this same ring is also present when performing the experiment on a sample which is prepared in the P state. This same variation between pulses is also the main cause of the relatively large error bar, as well as the apparent zero crossing and subsequent rise of the fluence gap in these measurements.

SUPPLEMENTARY NOTE 4: DISTINGUISHING BETWEEN SWITCHED STATES IN KERR MICROSCOPY IMAGES

The different contrast levels in the Kerr microscopy images in this work have thus far been labelled as different magnetization configurations. In this section we show how we determine which contrast level corresponds to which magnetization state.

In Supplementary Figure 4a we show Kerr microscopy images of a sample that has been prepared in the AP^+ state which has been exposed to a train of laser pulses (left, green border) and a single laser pulse (right, blue border). After exposure with a train of laser pulses, a complex multidomain magnetization state appears due to excessive heating of the sample. The energy of the laser pulses used here was high enough that both the reference and free layer have been thermally demagnetized by the pulse train. Therefore both states of both layers, and consequently all four possible magnetization configurations, are present in this area. This can be seen directly in the four different contrast levels that can be distinguished. In the case of exposure with a single laser pulse, only two total contrast levels are present.

In Supplementary Figure 4b we present histogram data of the gray values of all pixels in both images of Supplementary Figure 4a, after application of a slight Gaussian blur to reduce noise. Here, gray values of 0 and 255 are black and white, respectively. In the area that has been exposed to a train of laser pulses, four peaks in gray value are indeed present, which correspond with the four possible magnetization states. Conversely, for exposure with a single pulse only two peaks are present. We can now match the four contrast levels with their magnetization states by making use of the MOKE hysteresis loop presented in Fig. 1a of the main text. As mentioned previously, the sample has been prepared in the AP^+ state. This contrast level has the largest area, and therefore corresponds to the highest peak in Supplementary Figure 4b, or the second highest gray value. Second, the P states have the highest total Kerr rotation, and are therefore represented by the darkest and lightest levels. The settings of the Kerr microscope were chosen such that lighter contrast corresponds to higher positive Kerr rotation, which means the lightest (darkest) contrast level corresponds to the P^+ (P^-) state. The final remaining state, which has the second lowest gray value, is then the AP^- state.

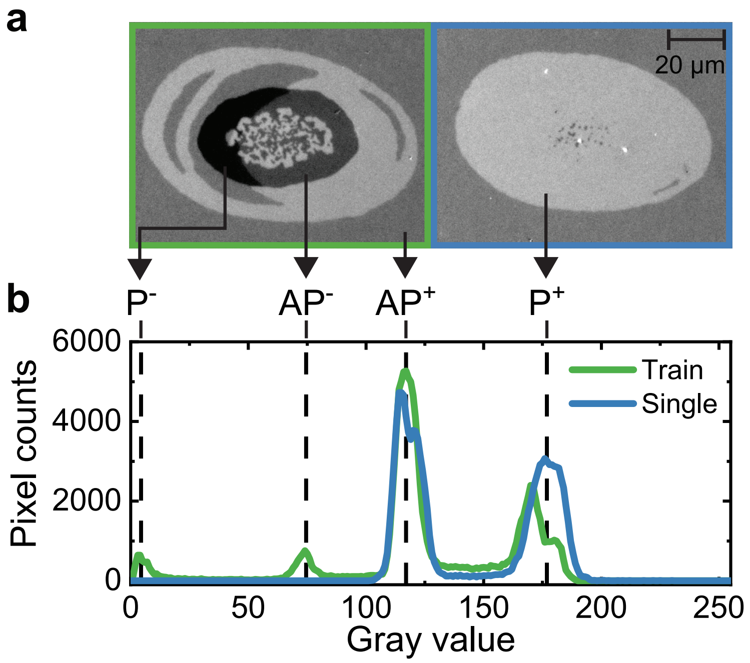

Supplementary Figure 4. Distinguishing between magnetization states. (a) Kerr microscopy images of a typical $(Co/Ni)_{x4}/Co/Cu/Co/Gd$ sample that has been exposed to a train of fs laser pulses (left) and a single laser pulse (right). (b) Histogram data of the gray values of each pixel in blurred versions of the images in (a). Four distinct contrast levels are observed and labelled with their corresponding magnetization states.

Here it is also clear that after a single pulse, only the free layer switches. When starting from a (+)-state, the appearance of a (-)-state is found only to occur after exposure to a train of laser pulses or a single laser pulse with high energy, both of which can lead to demagnetization of the reference layer. We note that the differences in gray value between the states do not precisely match with the steps found in the MOKE hysteresis loop. This is likely due to the use of white light in Kerr microscopy, as opposed to red laser light (632 nm) in the MOKE measurements, leading to different penetration depths and relative sensitivities to each of the magnetic layers.

REFERENCES

[1] "Filmetrics refractive index database," [Online; accessed January 9th 2020].

[2] S. Iihama, Y. Xu, M. Deb, G. Malinowski, M. Hehn, J. Gorchon, E. E. Fullerton, and S. Mangin, "Single-shot multi-level all-optical magnetization switching mediated by spin transport," Advanced Materials **30**, 1804004 (2018).

[3] M. Isasa, E. Villamor, L. E. Hueso, M. Gradhand, and F. Casanova, "Temperature dependence of spin diffusion length and spin hall angle in au and pt," Physical Review B **91**, 024402 (2015).
